# SP8 Promotes an Aggressive Phenotype in Hepatoblastoma via FGF8 Activation

**DOI:** 10.3390/cancers12082294

**Published:** 2020-08-15

**Authors:** Alexandra Elisabeth Wagner, Thomas Schwarzmayr, Beate Häberle, Christian Vokuhl, Irene Schmid, Dietrich von Schweinitz, Roland Kappler

**Affiliations:** 1 Department of Pediatric Surgery, Dr. von Hauner Children’s Hospital, Ludwig-Maximilians-University, 80337 Munich, Germany; Alexandra.Wagner@med.uni-muenchen.de (A.E.W.); beate.haeberle@med.uni-muenchen.de (B.H.); dietrich.schweinitz@med.uni-muenchen.de (D.v.S.); 2 Institute of Human Genetics, Helmholtz Center Munich, 85764 Neuherberg, Germany; thomas.schwarzmayr@gmx.at; 3 Institute of Pathology, University Hospital Bonn, 53127 Bonn, Germany; Christian.Vokuhl@ukbonn.de; 4 Department of Pediatrics, Division of Pediatric Hematology and Oncology, Dr. von Hauner Children’s Hospital, Ludwig-Maximilians-University Munich, 80337 Munich, Germany; Irene.Schmid@med.uni-muenchen.de

**Keywords:** hepatoblastoma, metastasis, SP8 transcription factor, fibroblast growth factor 8, mithramycin A

## Abstract

Hepatoblastoma (HB) is the most common malignant liver tumor in childhood and it generally has a good prognosis. However, if associated with aggressive metastatic disease, outcome is still poor. The molecular mechanisms leading to metastatic spread in HB patients are still unknown. By combining RNA-sequencing and a genome-wide methylome analysis, we identified the transcription factor SP8 and the growth factor FGF8 among the most strongly upregulated genes in metastatic HB cases, with a concomitant robust demethylation of the respective promoter regions. Of note, high expression of both candidates was associated with the aggressive C2 subtype of the 16-gene signature and poor survival. Chromatin immunoprecipitation revealed a direct transcriptional regulation of FGF8 through binding of SP8 to the *FGF8* promoter. Gain- and loss-of-function experiments proved promoting effects of SP8 on motility, self-renewal, migration, and the invasive potential of HB cells. Moreover, stable overexpression of SP8 in Hep3B cells resulted in the acquisition of a mesenchymal phenotype and a strong upregulation of epithelial-mesenchymal transition-associated genes. Using KRAB-mediated CRISPR-dCas9 interference directed against FGF8, we could show that FGF8 is essential for the SP8-mediated aggressive tumor behavior. Treatment of HB cell lines with the pan SP family inhibitor mithramycin A resulted in a significant inhibition of their clonogenic growth. In summary, we identified SP8 and FGF8 as key players in aggressive traits of HB and propose SP8 inhibiting drugs as a new effective treatment strategy especially for metastatic tumors.

## 1. Introduction

Hepatoblastoma (HB) is the most common pediatric liver tumor [1] with an annual incidence of 1.2 cases per million children per year in Europe and is most commonly diagnosed under the age of three years [2]. It is believed to arise from hepatic precursor cells undergoing malignant transformation during embryonic development [3]. For patients with standard-risk HB, the prognosis is favorable with a 3-year overall survival rate of 95%, if a complete surgical resection of the tumor can be achieved, either with or without chemotherapy [4]. However, for high-risk HB patients, prognosis is worse and the 3-year overall survival rate shrinks to 69% [5]. To improve risk-stratification, the Children’s Hepatic tumors International Collaboration (CHIC) globally pooled and analyzed clinical data from 1,605 HB cases [6]. They found that one of the main prognostic factors for high-risk HB was the existence of metastases [7].

The ability of tumor cells to spread to distant organs is mainly dependent on the so-called epithelial-mesenchymal transition (EMT), a reversible process in which epithelial cells acquire a mesenchymal phenotype by changing their morphology, cellular architecture, adhesion, and migratory capacity [8,9]. Cells that undergo EMT obtain self-renewing traits as described for stem cells and cancer stem cells, thereby enabling disseminated cells to serve as founders of metastatic colonies [10]. However, the molecular basis for these devastating properties in HB remains elusive.

Over the last few decades, several different approaches have been employed to elucidate the genomic profile of HB. This has identified a high prevalence of *CTNNB1* mutations in up to 90% of HB cases [11,12,13]. However, the general mutation rate of HB is very low, with only 3.9 mutations per tumor on average [14], which suggests mechanisms other than DNA mutation to cause metastatic spread. In this study, we used an integrated omics approach, combining transcriptional profiling and genome-wide methylation analysis to study differences between metastasized and non-metastasized HB genomic profiles. This allowed the identification of the zinc-finger transcription factor SP8 as a major factor involved in metastatic traits in malignant childhood liver tumors and revealed FGF8 as the main mediator of the SP8-induced aggressive tumor phenotype. Thus, we propose a close relationship between SP8, FGF8, and metastatic behavior in HB, suggesting an important role of the SP8-FGF8 axis in the progression of malignant childhood liver tumors.

## 2. Results

### 2.1. SP8 and FGF8 Are Overexpressed in HB with Metastasis and Poor Prognosis

**Figure 1:**
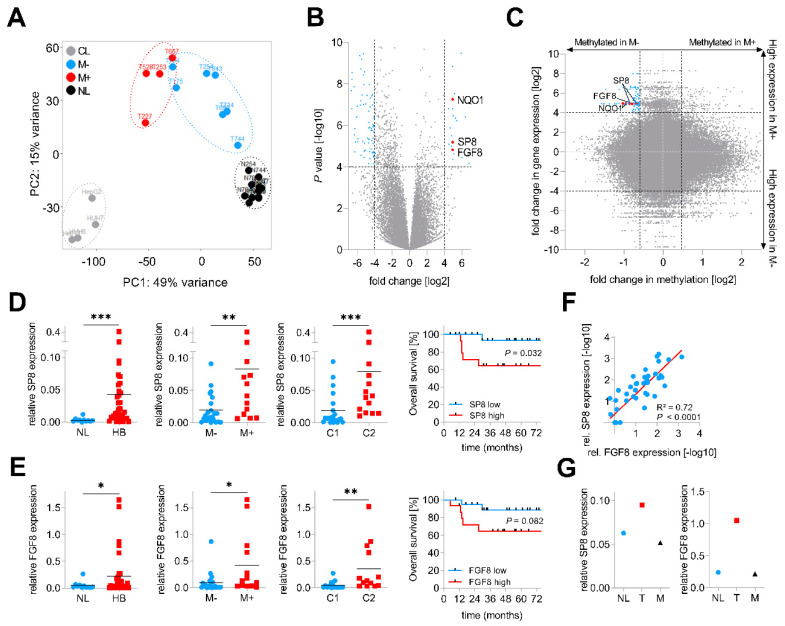
Identification of SP8 as a metastasis-associated marker of hepatoblastoma (HB). (**A**) principal component analysis of RNA sequencing-based expression data from 11 normal liver samples (NL, depicted in black), 7 non-metastasized (M−, in blue), 4 metastasized (M+, in red) primary tumors and 4 liver tumor cell lines (CL, in grey); (**B**) volcano plot displaying genes that are differentially expressed between M+ and M− tumors. Each gene is plotted with log2-fold expression change on the x-axis and negative log10 *p*-value on the y-axis. Genes with an absolute log2-fold change >4 and a *p*-value < 0.0001 are shown in blue. Candidate genes *NQO1*, *SP8* and *FGF8* are highlighted in red; (**C**) scatterplot with all CpG probes showing the correlation of promoter methylation and RNA expression between the M+ and M− subgroup. Genes with a fold change in methylation ≤ −1.5 and log2-fold expression change ≥4 are indicated in blue. CpG probes associated with SP8, FGF8 and NQO1 are indicated in red. (**D**) *SP8* and (**E**) *FGF8* expression levels were measured by Q-PCR, normalized to the house-keeping gene *TBP*, and categorized into NL and HB specimens, M− and M+ tumors, and the C1 and C2 subtype of the 16-gene signature [11]. Statistical significance of all experiments was calculated using the Mann–Whitney test (*p* < 0.05). Overall survival was calculated as time from diagnosis to death of the disease and is plotted for 35 HB patients. Statistical significance was calculated using the Mantel–Cox test; (**F**) correlation of *SP8* and *FGF8* expression in 35 tumor and 11 normal liver samples. Dots represent −log10 values of relative *SP8* expression plotted versus −log10 values of relative *FGF8* expression; (**G**) *SP8* and *FGF8* expression measured by Q-PCR and normalized to the house-keeping gene *TBP* in a patient with surgically removed metastasis (M) and primary tumor (T) with adjacent normal liver (NL). * *p* < 0.05, ** *p* < 0.01, *** *p* < 0.001.

In order to identify genes involved in metastatic spread, we performed RNA-sequencing of four primary hepatoblastomas with metastasis (M+) and seven primary hepatoblastomas without metastasis (M−), 11 matching normal liver (NL) specimens and four liver tumor cell lines (CL). Principal component analysis of the top 2,000 genes with the highest row variance revealed well-separated clusters for the NL samples and the CL and a more dispersed subcluster for the tumor samples, which could be separated into M− and M+ tumors (Figure 1A). By comparing global gene expression patterns between the two subgroups M+ and M−, we identified 24 upregulated and 93 downregulated genes (Figure 1B, Table S1). Among the top hits, we found NAD(P)H quinone dehydrogenase 1 (*NQO1*), a gene that was recently identified by our group as an important factor for aggressive tumor behavior and poor prognosis in HB [13]. In addition to *NQO1*, we also identified two new candidates, namely the Sp8 transcription factor (*SP8*) and the fibroblast growth factor 8 (*FGF8*), with highly increased transcription levels in tumors with metastasis.

In order to further characterize the origin of the transcriptional changes in HB, we paralleled this transcriptomic approach with a microarray-based DNA methylome analysis of the same 4 M+ and 7 M− primary tumor samples. Then, we crossed gene expression levels and the β-values of the corresponding CpG probes to identify new candidate genes with an inverse correlation between expression and methylation levels, which is fundamental for transcriptional regulation [15]. Strikingly, we found *NQO1* and the two candidate genes *SP8* and *FGF8* to harbor CpGs near their transcriptional start sites with lower methylation levels in M+ than in M− tumors (Figure 1C).

Next, we assessed the clinical relevance of both new candidates by measuring the gene expression levels of *SP8* and *FGF8* in an enlarged cohort of 35 HB samples and 11 normal liver specimens via Q-PCR analysis and then associating expression with clinicopathological features such as gender, age at diagnosis, histology, metastasis, vascular invasion, multifocality, PRETEXT stage, and outcome. Here, we could not only show that the mRNA levels of *SP8* and *FGF8* were generally increased in HB samples compared to normal liver, but also corroborated the RNA-sequencing results of a selective high *SP8* and *FGF8* expression in HB with metastasis (Figure 1D,E). In addition, high expression levels of both genes were significantly associated with the aggressive C2 subtype of the 16-gene signature established by Cairo et al. [11] (Figure 1D,E). Moreover, high expression levels were also prognostic for poor survival, although this was not significant for *FGF8* (Figure 1D,E). All other clinicopathological features were not significantly associated with *SP8* and *FGF8* expression (data not shown). Interestingly, there was a strong positive correlation of the *FGF8* and *SP8* expression (Figure 1F). Of note, we had one patient with non-metastatic HB at the initial diagnosis, but showed high *SP8* and *FGF8* levels in the primary tumor suggestive of metastatic disease (Figure 1D,E), who intriguingly developed a lung metastasis eight months after tumor resection. We could show that *SP8* and *FGF8* expression was increased only in the primary tumor, but not in the metastasis, as compared to adjacent normal liver (Figure 1G). However, as we have analyzed only one paired primary/metastatic tumor, the underlying mechanism for *SP8* and *FGF8* expression returning to normal in the metastasis, remains uncertain. Overall, these data suggest that SP8 and FGF8 play an important role in aggressive, metastatic HB with poor outcomes.

### 2.2. SP8 Transcriptionally Activates FGF8 and Promotes Motility, Invasiveness, and Self-Renewal of Hepatoma Cells

**Figure 2:**
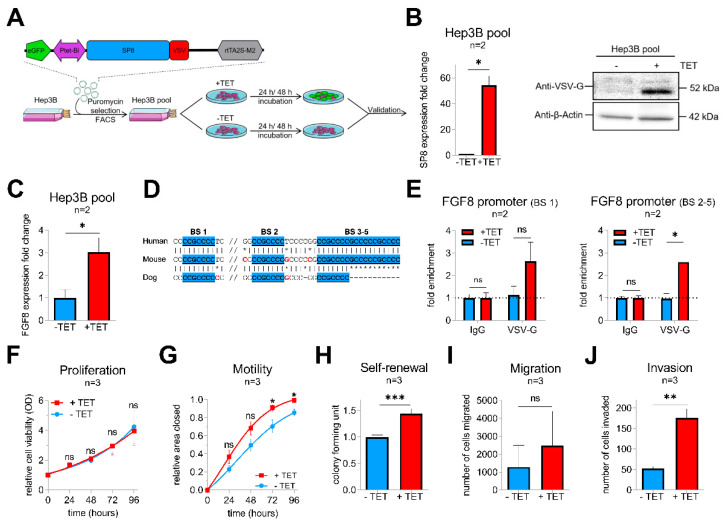
(**A**) schematic workflow of the validation approach of SP8 function in a Hep3B cell pool stably transfected with a TET-inducible SP8-eGFP-expression vector. Transfected cells were selected by puromycin treatment and enriched by fluorescence-assisted cell sorting (FACS). (**B**) SP8 overexpression 24 h after TET-induction was recognized by eGFP detection upon fluorescence microscopy (green cells) and proven on the RNA and protein level using Q-PCR and Western blotting, respectively. (**C**) *FGF8* expression levels after stable SP8-induction in Hep3B pool cells for 48 h was measured by Q-PCR (normalized to *TBP*). (**D**) schematic overview of the putative SP8 binding sites one to five upstream of the *FGF8* promoter predicted from the SP1-binding motif (GGGGCGG or CCGCCCC), highlighted in blue. (**E**) Chromatin immunoprecipitation was performed with Hep3B pool cells 48 h after TET-induction using an anti-VSV-G antibody or IgG control. Enrichment of VSV-SP8 binding on DNA fragments containing the putative SP8 binding site (BS) 1 (left panel) and BS2-5 (right panel) versus IgG background is plotted. Assays for (**F**) proliferation, (**G**) motility, (**H**) self-renewal, (**I**) migration and (**J**) invasion were conducted to validate tumor cell characteristics of TET-induced Hep3B cells. Means ± SEM were calculated from the indicated number of independent experiments. Statistical significance of all experiments was calculated using Mann–Whitney test (*p < 0.05, ** *p* < 0.01, *** *p* < 0.001).

To get more insights into the functional consequences of SP8 overexpression, we generated a stable pool of SP8-expressing Hep3B liver tumor cells by introducing the tetracyclin (TET)-inducible episomal vector system pRTR containing the reporter eGFP and the VSV-tagged SP8 cDNA (Figure 2A). Upon TET-induction, we could establish a significant upregulation of SP8 on the RNA level and the SP8-VSV fusion protein, as detected by the VSV-G tag (Figure 2B). Moreover, robust SP8 expression resulted in a significant upregulation of *FGF8* expression (Figure 2C).

To explore if FGF8 is a direct target of SP8, we scanned the *FGF8* promoter region for SP8 binding sites [16]. Interestingly, we identified five putative SP8 binding sites, which are highly conserved between mice and men (Figure 2D). Chromatin immunoprecipitation of Hep3B pool cells paired with Q-PCR then proved direct binding of SP8 to the *FGF8* promoter region at binding site 1 and 2–5 (Figure 2E), suggesting FGF8 as a direct target of the transcription factor SP8 and thereby explaining the close correlation of their expression in the primary tumors.

Next, Hep3B pool cells were further tested for their tumoral behavior upon SP8 induction. In proliferation assays, SP8-induction had no impact on growth rates of the tumor cells over time compared to non-induced cells (Figure 2F). However, wound-healing assays revealed that SP8-induction significantly promoted motility of Hep3B cells (Figure 2G) after 72 h and 96 h. Furthermore, colony formation assay showed significantly more colonies in SP8-induced cells (Figure 2H). In Boyden chamber assays, we found that SP8 overexpression also promoted cell invasion, but not migration (Figure 2I,J).

### 2.3. SP8 Leads to the Acquisition of EMT in Hep3B Cells

**Figure 3:**
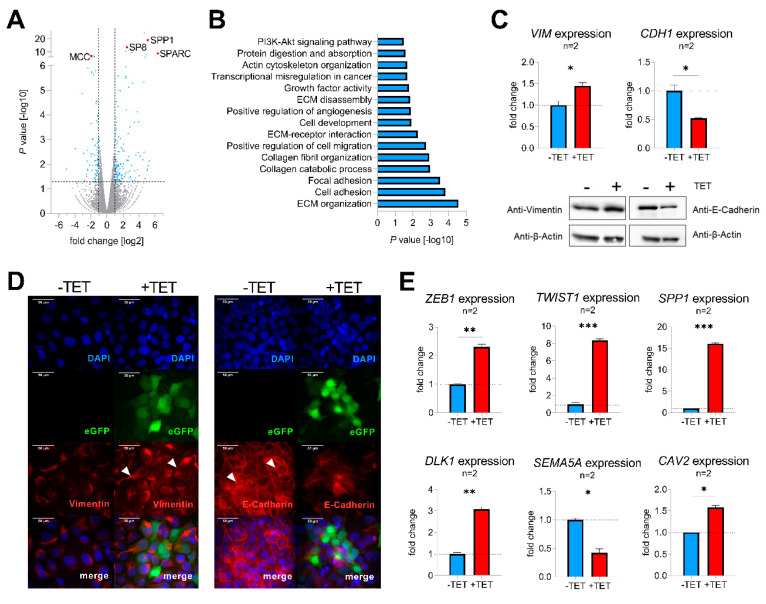
(**A**) volcano plot displaying genes that are differentially expressed between SP8-induced and non-induced Hep3B pool cells. Each gene is plotted with log2-fold expression change on the *x*-axis and negative log10 *p*-value on the y-axis. Genes with an absolute log2-fold change > 1 and a *p*-value < 0.05 are shown in blue. Candidate genes *SP8, SPP1, SPARC,* and *MCC* are highlighted in red; (**B**) functional annotation of differentially expressed genes between SP8-induced and non-induced Hep3B pool cells with a log2 fold change >1 and a *p*-value < 0.05; (**C**) detection of vimentin (VIM) and E-cadherin (CDH1) mRNA (upper part) and protein levels (lower part) in Hep3B pool cells 144 h after TET-induction by means of Q-PCR and Western blotting, respectively. Immunodetection of β-actin was used to standardize for equal protein loading. Representative Western blot images from three independent experiments are given. (**D**) Immunofluorescent staining (depicted in red) of vimentin (left panel) and E-cadherin (right panel) of Hep3B cells 144 h after TET-induction. Positively transfected cells expressing eGFP are depicted in green. DNA was counter-stained with DAPI (depicted in blue). Scale bar indicates 50 µm. (**E**) Expression of the EMT markers *ZEB1, TWIST1, SPP1, DLK1, CAV2*, and the epithelial marker *SEMA5A*, as determined by Q-PCR. Expression values are normalized to the house-keeping gene *TBP* and means ± SEM were calculated from the indicated number of independent experiments. Statistical significance of all experiments was calculated using a *t*-test (*p < 0.05, ** *p* < 0.01, *** *p* < 0.001).

To identify molecular mechanisms of SP8-mediated aggressive tumor behavior, TET-induced and non-induced Hep3B pool cells were subjected to RNA-sequencing, revealing 235 differentially expressed genes with a fold change > 2 and *p*-value < 0.05 (Figure 3A, Table S2). Two of the top upregulated genes were secreted protein acidic and rich in cysteine (SPARC; also known as osteonectin) and secreted phosphoprotein 1 (SPP1; also named osteopontin), known drivers of epithelial-mesenchymal transition (EMT) by inhibiting the cell-cell adhesion molecule E-cadherin (CDH1) [17,18]. Moreover, one of the top downregulated genes was mutated in colorectal cancer (MCC), which enhances cell-cell adhesion through interacting with CDH1 [19]. In line with this, functional annotation of the detected candidate genes with DAVID revealed gene enrichment in extracellular matrix (ECM organization, ECM receptor interaction and ECM disassembly), cell adhesion, focal adhesion, and positive regulation of cell migration (Figure 3B), which suggested a general involvement of SP8 in EMT. As the upregulation of the mesenchymal marker vimentin (VIM) and downregulation of the cell adhesion marker CDH1 display the main mechanism underlying EMT [20], we checked both genes for their expression level after SP8 induction. Of note, we found an appropriate expression pattern of both markers on the RNA as well as the protein level (Figure 3C). Immunofluorescent staining of VIM and CDH1 validated induction of a mesenchymal phenotype in Hep3B cells, as indicated by the spindle-like morphology of induced cells compared to the more compact epithelial phenotype of non-induced cells (Figure 3D). In addition, other EMT marker genes like zinc finger E-box binding homeobox 1 (ZEB1), twist-related protein 1 (TWIST1), secreted phosphoprotein 1 (SPP1), delta-like 1 homolog (DLK1), semaphorin A (SEMA5A), and caveolin 2 (CAV2) were also found to be significantly modulated in SP8-induced cells (Figure 3E). In summary, these results indicate that SP8 overexpression leads to the acquisition of a mesenchymal phenotype.

### 2.4. FGF8 Is Essential for SP8-Mediated Aggressive Tumor Behavior

**Figure 4:**
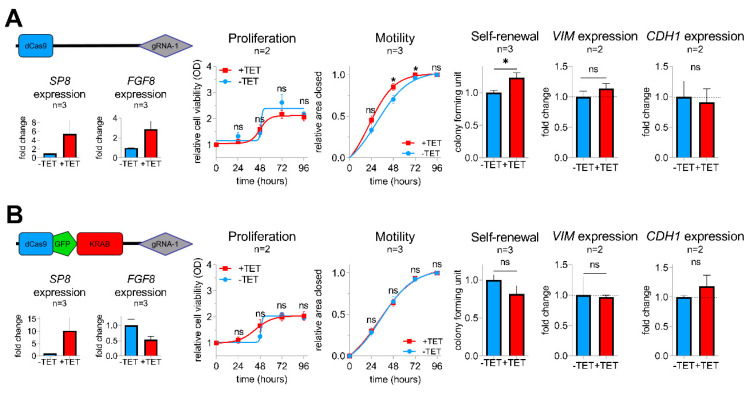
Vectors for dCas9 (**A**) and dCas9-GFP-KRAB (**B**) expression used for the CRISPR-Cas9 interference-mediated *FGF8* knockdown with guide RNA 1 directed against *FGF8*. *SP8, FGF8, VIM,* and *CDH1* mRNA expression was measured using Q-PCR and normalized to the housekeeping gene *TBP*. Proliferation, motility, and colony formation assays of TET-induced and non-induced Hep3B pool cells in the presence of either the pPlat-dCas9-gRNA-1 control vector (upper panel) or the knockdown mediating pPlat-dCas9-GFP-KRAB-gRNA-1 vector (lower panel). Means ± SEM were calculated from the indicated number of independent experiments. Statistical significance of all experiments was calculated using a t-test (* *p* < 0.05).

To determine whether FGF8 is required for the SP8-induced aggressive phenotype, we generated a sustained knockdown of FGF8 expression in SP8-induced Hep3B pool cells using a KRAB-mediated CRISPR-dCas9 interference approach [21], as described in the Supporting Experimental Procedures (Figures S1,S2,S6). As anticipated, control Hep3B pool cells containing an empty dCas9 control vector showed elevated levels of both *SP8* and *FGF8* after TET-induction and an increased motility and colony formation capacity, whereas proliferation stayed unchanged (Figure 4A). In contrast, the dCas9-GFP-KRAB fusion construct guided against FGF8 resulted only in elevated levels of *SP8*, but led to a marked knockdown of FGF8, which prevented the SP8-induced increase in cell motility and self-renewal (Figure 4B). Moreover, the slight SP8-induced increase and decrease of the EMT marker genes *VIM* and *CDH1* were reversed upon FGF8 knock-down, respectively. These data validate FGF8 as the major mediator of the SP8-induced aggressive phenotype in liver cancer cells.

### 2.5. SP8-Mediated Aggressiveness Is A General Trait in Liver Cancer Cells

To exclude that the observed effects of SP8 are only restricted to Hep3B cells, we additionally performed transient gain- and loss-of-function studies in the five liver cancer cell lines HepG2, Hep3B, HUH7, HUH6, and HepT1, which show either high (HepT1 and HUH6) or low levels (HepG2, Hep3B, HUH7) of endogenous *SP8* expression (Supporting Experimental Procedures Figure S3). The successful *SP8* modulation and the subsequent *FGF8* expression changes were validated by Q-PCR (Supporting Experimental Procedures Figure S4) and were similar to the Hep3B pool cells.

**Figure 5:**
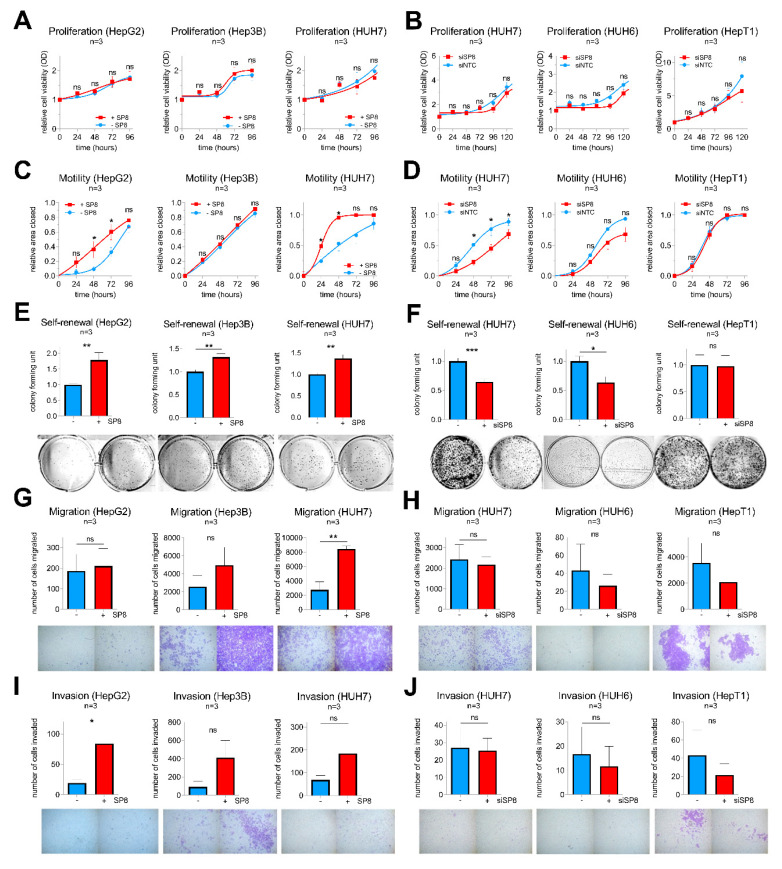
Gain- and loss-of-function studies in liver cancer cell lines after transfection with either (**A**,**C**,**E**,**G**,**I**) an empty control vector (−SP8) and a SP8 cDNA containing expression plasmid (+SP8), or (**B**,**D**,**F**,**H**,**J**) non-targeting siRNAs (siNTC) and a siRNA directed against *SP8* (siSP8), respectively. (**A**,**B**) Cell proliferation was measured at the indicated time points using MTT assays. (**C**,**D**) Cell motility was determined in scratch assays at the indicated time points. (**E**,**F**) Self-renewal was determined by counting colonies 10 days after transfection. Representative images of cell assays are shown below the graphs. (**G**,**H**) Migration was measured by counting cells that migrated through pores of Boyden chambers without matrigel coating 48 h after transfection. (**I**,**J**) Invasion was measured by counting cells that migrated through pores of Boyden chambers coated with matrigel 72 h after transfection. Means ± SEM were calculated from the indicated number of independent experiments. Statistical significance of all experiments was calculated using Mann–Whitney (* *p* < 0.05, ** *p* < 0.01, *** *p* < 0.001).

Consistent with the stable experiments mentioned above, SP8 modulation resulted in no difference in proliferation in all five cell lines (Figure 5A,B), but had a significant impact on motility in HepG2 and HUH7 cells (Figure 5C,D). Moreover, colony formation assays revealed a promoting effect of SP8 overexpression on self-renewal in HepG2, Hep3B, and HUH7 cells (Figure 5E), and this effect could be reversed by *SP8* knockdown in HUH7 and HUH6 cells to about 60% of the colony formation capacity of the control cells (Figure 5F). Furthermore, promoting effects on migration and invasion upon SP8 overexpression were evidenced in HUH7 (Figure 5G) and HepG2 cells (Figure 5I), respectively. Although the effects on migration and invasion upon *SP8* knock-down were in general quite low and not significant, due to the already weak migration and invasion capacities of the cell line itself, there was a trend toward reduced migratory and invasive properties in knockdown cells (Figure 5H,J). Overall, these data suggest that SP8 plays an important role on cell motility, invasiveness, and self-renewal, thereby leading to a more aggressive behavior of liver tumor cells.

### 2.6. SP Family Inhibition as A Potential Therapeutic Option for Aggressive Hepatoblastoma

**Figure 6:**
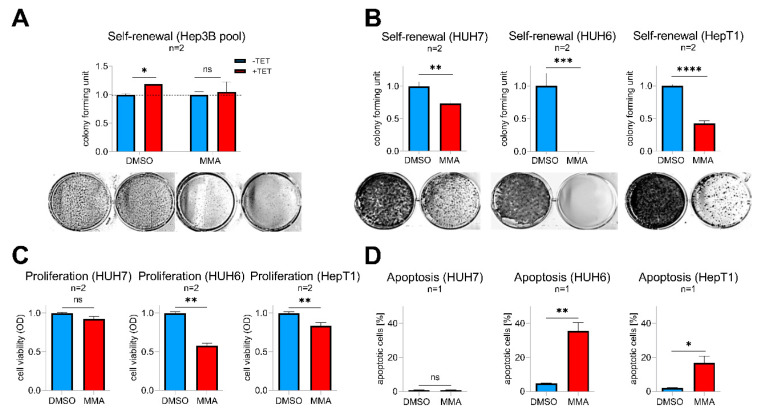
(**A**) Hep3B pool cells were treated with 10 nM mithramycin A (MMA) or DMSO as a control with (+TET) or without (−TET) SP8-induction. (**B**–**D**) HUH7, HUH6, and HepT1 cells were treated with 10 nM (HUH7 and HUH6) or 30 nM (HepT1) MMA or DMSO as a control. (**A**,**B**) Self-renewal was determined by counting colonies 10 days after MMA treatment and results normalized to 1. Representative images of cell assays are shown below the graphs. (**C**) Proliferation and (**D**) apoptosis assays were conducted 48 h after MMA treatment. Means ± SEM were calculated from the indicated number of independent experiments. Statistical significance of all experiments was calculated using t-test (* *p* < 0.05, ** *p* < 0.01, *** *p* < 0.001, **** *p* < 0.0001).

Mithramycin A (MMA), a FDA-approved anticancer drug, is a potent pan SP transcription factor family inhibitor that is able to prevent their binding to GC-rich gene promoters [22]. As a proof-of-concept, we first wanted to see if MMA is able to abrogate SP8-induced effects in Tet-induced Hep3B pool cells. Indeed, MMA-treated cells showed a complete inhibition of the SP8-induced increase in colony formation compared to solvent-treated cells (Figure 6A). By testing varying doses of 1–100 nM MMA on SP8-high expressing hepatoma cell lines, we found that doses down to 10 nM (HUH7 and HUH6) and 30 nM (HepT1) significantly reduced self-renewal (Figure 6B). Interestingly, these effects were much stronger than the ones observed after specific SP8 knock-down (Figure 5), thus leading us to further investigate the consequences of MMA treatment in more detail. As expected, we found that MMA downregulated *SP8* in all three cell lines (Supporting Experimental Procedures Figure S5). However, MMA also led to a marked inhibition of viability through a concomitant induction of apoptosis in the two hepatoblastoma cell lines HUH6 and HepT1, but not in the HCC cell line HUH7 (Figure 6C,D). Interestingly, HUH7 cells did also not respond to the MMA treatment with a reduced *FGF8* expression (Supporting Experimental Procedures Figure S5). Therefore, we conclude that MMA due to its pleiotropic effect on multiple SP transcription factors might present an effective treatment option of high-risk HB by combining both SP8-mediated and cytotoxicity-triggered effects. 

## 3. Discussion

Complete surgical resection remains the treatment option of choice for achievement of a cure in HB patients, and metastasis is considered as the major barrier to attainment of this goal [6]. Knowledge of the molecular mechanisms that causes metastatic spread in HB patients would help with approaches to clinically manage patients with metastasis according to their individual risk profile and with detection of metastases at an earlier stage. However, due to the rare occurrence of this tumor, there is only limited data available on the important players in metastatic HB. In this study, we combined RNA-sequencing and genome-wide methylome analysis to allow for the in-depth characterization of metastasized HB samples. We thus identified the transcription factor SP8 and the growth factor FGF8 as two of the most strongly upregulated genes in metastatic HB cases. This finding was accompanied by hypomethylation of CpG probes close to the promoter regions of these genes. More importantly, high SP8 and FGF8 expression was associated with the presence of the aggressive C2 subtype of the 16-gene signature [11] and poor outcome, and could be linked in vitro to aggressive traits such as cell motility, self-renewal, migration, and invasion. Consequently, these effects could be successfully abrogated by downregulating FGF8, which we identified as a direct transcriptional target of SP8.

SP8 belongs to the closely related family of SP zinc-finger transcription factors that are not only critical players during embryonal and postnatal development, but are also often found to be upregulated in many cancers [23]. Although high expression of SP family members has generally been associated with advanced tumor stage, an invasive potential, metastasis, and poor patient survival in many cancers [23,24], the exact role of SP8 has remained unclear until now. However, a first study on its promoting role in the migration of cortical interneurons has just recently been published [25], which suggests SP8-induced mechanisms to be also involved in adverse cellular processes in cancer. Indeed, we found that one such mechanism could be the transcriptional upregulation of *FGF8*, although the relationship between SP8 and FGF8 might be more complex, since upregulation of *FGF8* could only be detected 48 h, but not 144 h post-induction in our Hep3B pool cells, which might be explained by an existing feedback loop [16]. Nevertheless, upregulation of *FGF8* has been described in many tumor types, such as breast cancer [26], colorectal cancer [27], prostate cancer [28], and most interestingly hepatocellular carcinoma [29]. Clinically, it has been associated with advanced tumor stage, lymph node metastasis, and decreased patient survival in prostate and colorectal cancer [27,30]. These adverse characteristics mainly depend on the biological role described for FGF8, which comprises the induction of the migration and invasion capacity of cancer cells and has been tested both in vitro and in vivo in colorectal and breast cancer [27,31]. Consequently, preclinical testing of a neutralizing antibody to FGF8 significantly inhibited growth of prostate cancer cells in vitro [30] and of mammary tumors in nude mice [32]. Intriguingly, bone metastases from prostate cancer are positive for FGF8 and its ectopic expression increased the growth of prostate cancer cells as intratibial tumors [28]. Collectively, these studies convincingly underscore the importance of SP8-induced FGF8 in metastatic disease, as we have shown here for HB.

As our functional experiments highlighted the significance of SP8 in promoting cell motility, self-renewal, migration, and invasion by activating FGF8, it was not surprising that SP8-induced Hep3B pool cells developed mesenchymal traits and exhibited a gene expression pattern suggestive of EMT. It is well known that EMT induction requires a set of transcription factors such as ZEB1 and TWIST1 together with several other factors such as FGFs to ultimately repress the cell adhesion mediator CDH1 and to upregulate the intermediate filament VIM [20]. Accordingly, SP8-overexpressing Hep3B pool cells showed induction of ZEB1, TWIST1 and VIM, as well as downregulation of CDH1. They also showed upregulation of other EMT promoting genes such as osteonectin, osteopontin, and DLK1 [17,18,33]. These results, along with previous data on the FGF8-induced acquisition of EMT in colorectal cancers [27], clearly indicate that the SP8/FGF8 axis contributes to metastasis by inducing EMT.

Another intriguing finding of our study is the strong anti-tumorigenic effect of the SP transcription factor inhibiting drug MMA [22]. MMA has already been described as a potent drug in preclinical trials involving prostate [34], breast [35], and colon cancer [36], in which doses in the nanomolar range, comparable to our concentrations, exhibited profound anti-tumorigenic effects. Moreover, MMA has also been shown to efficiently inhibit EMT [37]. Although MMA is not a specific SP8 inhibitor and thus the dramatic decline in self-renewal capacity of the liver tumor cells of our study cannot be ascribed to SP8 inhibition alone, our data clearly support the use of SP8 inhibition to interfere with metastatic liver tumors. However, as the broad and unspecific downstream effects of MMA result in a strong toxicity in clinical studies [38], the generation of new more targeted MMA analogues such as MTM-SDK and MTM-SK with improved pharmacological and toxicological profiles [39] and the evaluation of combination therapies with the current standard of care therapies (such as doxorubicin in HB [35]) are promising future directions.

However, this study also has several limitations. First, some parts of the experimental setup have only been tested with a low number of replicates and might therefore be based on weak statistics. However, the main conclusions have been deduced from the gain- and loss- of function assays that were conducted in three independent experiments in five different cell lines, of which Hep3B was tested in a transient and stable transfected condition. Second, we have chosen the hepatocellular carcinoma cell line Hep3B because of its low endogenous *SP8* expression as a study model for inducible SP8 gain-of function experiments, which might display some tumor properties distinct from HB. However, a recent report described its differentiation level as “hepatoblast-like” and showed that Hep3B is closer related to HB cell lines than to “classical” HCC cell lines [40]. Third, additional important candidates that might also contribute to HB aggressiveness might go unnoticed from our RNA sequencing approaches on metastatic and non-metastatic tumors as well as SP8-induced genes in Hep3B pool cells. Thus, future studies are warranted to decipher the role of well-known drivers of EMT such as SPARC, SPP1, and MCC, or other potential players in primary HB with metastasis that have to be retrieved from our supplemental data (Table S1).

## 4. Materials and Methods

### 4.1. Human Subjects

A total of 35 childhood liver tumor samples were obtained from patients undergoing surgical resection in our department after receiving chemotherapy according to the German HB99 study protocol [41]. Associated normal liver specimens were available from eleven of these patients. Written informed consent was obtained from each patient and the study protocol was approved by the Ludwig-Maximilians-University Ethics Committee in Munich (project code: 431-11). Summarized patient characteristics are shown in Table S3. Assignment of tumors to either C1 or C2 according to the 16-gene signature was accomplished by using quantitative polymerase chain reaction and the BRB ArrayTools software class prediction tool (http://linus.nci.nih.gov/BRB-ArrayTools.html) as described previously [11].

### 4.2. Cell Culture

We used the three HB cell lines HUH6 (Japanese Collection of Research Bioresources, Osaka, Japan), HepG2 (ATCC, Manassas, VA, USA), and HepT1 [42], as well as the two hepatocellular carcinoma cell lines Hep3B and HUH7 [43]. For optimizing CRISPR-dCas9 interference-mediated knockdown of FGF8, RMS13 (ATCC, Manassas, VA, USA), cells were taken. All cell lines were maintained in RPMI 1640 supplemented with 10% (*v*/*v*) fetal bovine serum (FBS) and 1% Pen-Strep at 37 °C in a humidified atmosphere containing 5% CO_2_.

### 4.3. RNA Isolation

Total RNA was extracted from fresh-frozen tumor samples, healthy control tissues and cell lines in TRIzol (Invitrogen, Karlsruhe, Germany), followed by phase separation with chloroform and isopropanol precipitation according to the manufacturer’s recommendations. Quantification and quality control of RNA samples was performed using a Nanodrop ND-1000 (Peqlab, Erlangen, Germany) and the 2100 Bioanalyzer (Agilent Technologies, Santa Clara, CA, USA) respectively.

### 4.4. RNA Sequencing

In addition, 1 µg total RNA from each of 11 HB samples, 11 matching normal liver (NL) samples and 4 liver tumor cell lines was used to enrich coding transcriptomes with the TruSeq non-stranded RNA v2 kit (Illumina, San Diego, CA, USA). Sequencing was done on a HiSeq2500 system (Illumina) as 100 bp paired-end runs generating 35-83 million mapped reads. The STAR aligner v 2.4.2a [44] with modified parameter settings (--twopassMode=Basic) was used for split-read alignment against the human genome assembly hg19 (GRCh37) and UCSC known gene annotation. HTseq-count v0.6.0 [45] was used to quantify the number of reads mapping to annotated genes. Read counts obtained from RNA-seq data were normalized and analyzed for differential expressed genes between metastasized and non-metastasized HB by using the Bioconductor package DESeq2 [46]. Data are available at Gene Expression Omnibus (accession number GSE151347).

### 4.5. DNA Methylation Array Analysis

Bisulfite-converted DNA from each of 11 HB samples were hybridized to Human Methylation 450 BeadChip microarrays (HM450K, Illumina) following the Illumina Infinium HD methylation protocol. All data processing steps were carried out using the minfi package [47]. First, probes were normalized using stratified quantile normalization preprocessing implemented in the preprocessQuantile function [48]. Then, probes located in SNPs (17,541 probes) as well as probes located on the X and Y chromosomes (11,458 probes) were excluded from downstream analyses resulting in a total of 456,513 probes for each of the 11 HB samples. Probe annotation was performed using the manifest file with UCSC version hg19 of the human reference genome. The methylation levels for each CpG probe were provided as β-values ranging from 0 to 1 (0 indicating unmethylated CpGs and 1 indicating fully methylated CpGs). Lastly, to correct β-values for known batch effects the ComBat function implemented in the SVA package [49] was applied.

### 4.6. Integration of Gene-Level Differential Methylation and Expression Analyses

CpG probes associated with a specific gene were extracted from the manifest file and compared to expression levels of the respective gene between metastatic and non-metastatic HB. Briefly, for each CpG probe, mean β-values were calculated for metastasized and non-metastasized HB cases and the log2-fold change in methylation between both groups extracted. The log2-fold change in the expression of each gene was calculated using DESeq2 and expression values aligned with matching CpG probes according to the manifest file. Each CpG probe was plotted with log2-fold change in methylation on the x-axis and log2-fold expression change on the y-axis.

### 4.7. Transfection and Generation of Stable Cell Lines

Detailed information on vector generation, transfection of plasmid-DNA and siRNA, and the generation of stable Hep3B cells can be found in the Supporting Experimental Procedures.

### 4.8. Quantitative Polymerase Chain Reaction (Q-PCR)

In addition, 2 µg of total RNA was reverse transcribed to cDNA using SuperScript™ II Reverse Transcriptase (Thermo Fisher Scientific, Waltham, MA, USA) and random hexamer primers (Roche, Mannheim, Germany). Samples were analyzed in duplicates by Q-PCR on a Master cycler ep gradient (Eppendorf, Hamburg, Germany) in 20 µL reaction volumes using gene specific primers (Table S4) and the SYBR-Green Mastermix (BioRad, Hercules, CA, USA) according to the supplier. Normalization was carried out with the TATA box binding protein (TBP) as a reference.

### 4.9. Western Blot Analysis

Cell lysates were obtained by using RIPA lysis buffer [25 mM Tris/HCl, pH 8.0, 150 mM NaCl, 1% Nonidet P-40, 1% (w/v) sodium deoxycholate, 0.1% SDS, 1x cOmplete Mini protease inhibitor cocktail tablet (Sigma-Aldrich, St. Louis, MO, USA)]. Lysates were centrifuged at 13,000 g for 30 min at 4 °C. Per lane, 25–50 µg of whole-cell lysate was separated on 4–20% SDS acrylamide gels and transferred to PVDF membranes (Bio-Rad, Hercules, CA, USA). For immunodetection, membranes were incubated with antibodies specific for the VSV-G tag (Sigma-Aldrich), β-Actin, Vimentin and E-Cadherin (all from Cell Signaling, Danvers, MA, USA). Signal detection from HRP-conjugated secondary antibodies was carried out with enhanced chemiluminescence (GE Healthcare, Chicago, IL, USA) and recorded with the Diana III chemiluminescence imager (Raytest GmbH, Straubenhardt, Germany).

### 4.10. Cell Viability Assay

Transient or stable transfected cells (5 × 10^3^) were plated in 96-well plates with a culture volume of 100 µL/ well. In addition, 10 µL of MTT solution I [3-(4,5-dimethylthiazol-2yl)-2,5-diphenyl tetrazolium bromide, 5 mg/ml in PBS, sterile-filtered] was added, and cells were incubated for 4 h at 37 °C. MTT solution II (10% SDS, 0.37% HCl) was then given to the wells and cells incubated overnight at 37 °C. Optical density was measured at 595 nm wavelength by using the GENios microplate reader (Tecan, Männedorf, Switzerland).

### 4.11. Wound Healing Assay

Furthermore, 0.1 × 10^6^ transient or stable transfected cells/ well were seeded into 12-well plates and grown to confluency. A wound of approximately 1 mm was inflicted to cell monolayers with a pipette-tip and cells allowed to close the wound for 96 h. Images were taken every 24 h with an Axiovert 40CFL microscope (Zeiss, Oberkochen, Germany) connected to a 450D camera (Canon, Tokyo, Japan), and the wound widths were measured and quantified using ImageJ (version, National Institute of Health, Bethesda, MD, USA).

### 4.12. Boyden–Chamber Assay

Transwell permeable supports (8 μm pore polycarbonate inserts; Corning Inc., Corning, NY, USA) were either coated with 50 µL of matrigel solution (1:1 dilution with RPMI 1640) to analyze invasion or used without coating to analyze migration, and added to 24-wells containing RPMI 1640 with 30% FCS as a chemoattractant. In addition, 1 × 10^5^ cells for invasion and 0.7 × 10^5^ cells for migration were seeded in RPMI 1640 without supplements in the upper compartment and allowed to migrate for 48 h (migration) or 72 h (invasion). Afterwards, the cells were fixed, permeabilized, and stained with 0.5% crystal violet solution. Cells in the upper compartment were removed with cotton swabs and the number of migrated cells assessed by documentation with an Axiovert 40CFL microscope (Zeiss) connected to a 450D camera (Canon). Images were analyzed with the ImageJ Cell Counter plugin (National Institute of Health).

### 4.13. Colony Formation Assay

In addition, 5 × 10^3^ cells/ 6-well plate were seeded and cultivated for 8–10 days at 37 °C. Afterwards, the cells were fixed, permeabilized and stained with 0.5% crystal violet solution. For quantification of colonies, pictures were taken using the GelJet Imager Version 2004 and analyzed with the ImageJ Cell Counter plugin (National Institute of Health).

### 4.14. Apoptosis Assay

Furthermore, 2 × 10^5^ cells/ 12-well plate were seeded and cultivated for 2 days with the addition of mithramycin A at the indicated concentrations or DMSO at 37 °C. Then, the cells were trypsinized, washed with PBS, resuspended in 300 µL Nicoletti buffer (0.1% Na_3_C_6_H_5_O_7,_ 50 µg/mL propidium iodide, 0.1% Triton X-100), and incubated for 2 h at 4 °C. Afterwards, cells were washed with PBS and resuspended in 300 µL PBS. The cells were analyzed and recorded by the flow cytometer BD LSRFortessa (BD, Franklin Lakes, NJ, USA) and the apoptotic cell fraction (sub-G1 peak) quantified using FlowJo (FlowJo LLC, Ashland, OR, USA).

### 4.15. Immunofluorescence

Cells were fixed in 3.7% paraformaldehyde/PBS for 15 min, permeabilized with 100% methanol for 10 min and blocked with 5% BSA and 0.3% Triton X-100 for 1 h. For the detection of Vimentin or E-Cadherin, the rabbit anti-Vimentin (D21H3) antibody and rabbit anti-E-Cadherin (24E10) antibody (both from Cell Signaling Technology) were used as primary antibodies, respectively, and Alexa Fluor555 conjugated goat anti-rabbit IgG (Invitrogen) was used as a secondary antibody. Images were captured on an Axiovert 200M microscope equipped with an AxioCam MRm and the AxioVision software (all from Zeiss).

### 4.16. Chromatin Immunoprecipitation

Stably transfected Hep3B cells were cross-linked by adding 37% paraformaldehyde/PBS to the medium to a final concentration of 1% and rotated for 10 min. The reaction was stopped with the addition of glycine to a final concentration of 125 mM. Cells were washed twice with PBS and lysed with cell lysis buffer [5 mM PIPES (pH 8), 85 mM KCl, 0.5% NP-40] for 10 min followed by nuclei lysis [1% SDS, 10 mM EDTA, 50 mM Tris-HCl (pH 8.1)] for 10 min on ice. Chromatin was sheared to an average size of 500 bp by the application of 1 µL micrococcal nuclease (100 U; Thermo Fisher Scientific) for 7 min at RT and sonication (6 × 15 sec on off cycle, 10% amplitude). After pre-clearing with protein G agarose beads (Sigma-Aldrich) for 1 h, lysates were rotated overnight at 4 °C with the polyclonal anti-VSV-G antibody (Sigma-Aldrich) or rabbit anti-mouse IgG (Abcam, Cambridge, United Kingdom) as a negative control. After several wash steps with low salt wash buffer [0.1% SDS, 1% Triton X-100, 2 mM EDTA, 20 mM Tris-HCl pH 8.1, 150 mM NaCl], high salt wash buffer [0.1% SDS, 1% Triton X-100, 2 mM EDTA, 20 mM Tris-HCl pH 8.1, 500 mM NaCl] and LiCl wash buffer [250 mM LiCl, 1% NP40, 1% DOC, 1 mM EDTA, 10 mM Tris-HCl pH 8.1] and bead elution with elution buffer [1% SDS, 100 mM NaHCO_3_], samples were subjected to overnight reversal of cross-linking with 5 M NaCl at 65 °C. DNA was purified using the PCR purification kit (Qiagen, Hilden, Germany) and subjected to Q-PCR amplification using FGF8 promoter-specific primers (Table S4).

### 4.17. Statistical Analysis

The data are presented either as dot plots or bar graphs, indicating the mean ± standard error of the mean (SEM). Statistical analysis was carried out with GraphPad Prism 8.2.1.0 software (GraphPad Software, San Diego, CA, USA) or analyzed with the statistics language R software version 3.5.1 and the Bioconductor suite [50]. Functional annotation was performed with the database for annotation, visualization and integrated discovery (DAVID) [51].

## 5. Conclusions

Due to the lack of knowledge on the molecular basis of metastatic spread in childhood liver cancer and thus targeted intervention strategies, hepatoblastoma patients with metastasis still face a poor outcome. In this work, we identified the transcription factor SP8 and the growth factor FGF8 among the most strongly upregulated genes in metastatic HB cases. Mechanistically, we could show that FGF8 is a direct target gene of SP8 by binding of SP8 to the FGF8 promoter. Of clinical relevance, high expression of both genes was associated with the aggressive C2 subtype of the 16-gene signature and poor survival. Functional analyses clearly indicated that elevated levels of SP8 induce aggressive traits of liver tumor cells by enhancing their motility, invasiveness, and self-renewal, as well as provoking epithelial-mesenchymal transition. Consequently, SP8-triggered cellular processes could be abrogated by inhibiting the downstream target FGF8. Altogether, these results underscore an important functional role of the SP8-FGF8 axis in the progression and metastasis of malignant childhood liver tumors. Moreover, SP8 and FGF8 might serve as prognostic biomarkers in metastatic HB that could indicate patients who would benefit from SP inhibiting drugs. Although the pan SP family inhibitor mithramycin A proved to be effective in our in vitro studies, the identification of more targeted approaches with improved pharmacological and toxicological profiles are warranted to bring SP8-FGF8 interference closer to the clinic.

## Supplementary Materials

The following are available online at https://www.mdpi.com/2072-6694/12/8/2294/s1, Table S1: DEGs HB-Tumors M+ vs. M−; Table S2: DEGs Hep3B pool TET+ vs. TET-; Table S3: Human Subjects; Table S4: Oligos and Primers; Figure S1: Schematic overview of the generated constructs; Figure S2: Validation of the pPlat-dCas9-GFP-KRAB construct; Figure S3: Endogenous expression levels of SP8 and FGF8 in liver tumor cell lines; Figure S4: Gain- and loss-of-function expression studies of SP8 and FGF8 in liver cancer cell lines; Figure S5: RNA expression of SP8 and FGF8 after MMA treatment; Figure S6: CRISPR-Cas9 interference testing.
