# Erratum: Wagner, A.E., et al. SP8 Promotes an Aggressive Phenotype in Hepatoblastoma via FGF8 Activation. *Cancers* 2020, *12*, 2294

**DOI:** 10.3390/cancers13030362

**Published:** 2021-01-20

**Authors:** Alexandra Elisabeth Wagner, Thomas Schwarzmayr, Beate Häberle, Christian Vokuhl, Irene Schmid, Markus Kaller, Heiko Hermeking, Dietrich von Schweinitz, Roland Kappler

**Affiliations:** 1 Department of Pediatric Surgery, Dr. von Hauner Children’s Hospital, Ludwig-Maximilians-University, 80337 Munich, Germany; Alexandra.Wagner@med.uni-muenchen.de (A.E.W.); beate.haeberle@med.uni-muenchen.de (B.H.); dietrich.schweinitz@med.uni-muenchen.de (D.v.S.); 2 Institute of Human Genetics, Helmholtz Center Munich, 85764 Neuherberg, Germany; thomas.schwarzmayr@gmx.at; 3 Institute of Pathology, University Hospital Bonn, 53127 Bonn, Germany; Christian.Vokuhl@ukbonn.de; 4 Department of Pediatrics, Division of Pediatric Hematology and Oncology, Dr. von Hauner Children’s Hospital, Ludwig-Maximilians-University Munich, 80337 Munich, Germany; Irene.Schmid@med.uni-muenchen.de; 5 Experimental and Molecular Pathology, Institute of Pathology, Ludwig-Maximilians-University Munich, 80337 Munich, Germany; markus.kaller@med.uni-muenchen.de (M.K.); heiko.hermeking@med.uni-muenchen.de (H.H.); 6 German Cancer Consortium (DKTK), Partner Site Munich, 80336 Munich, Germany; 7 German Cancer Research Center (DKFZ), 69120 Heidelberg, Germany

Two contributors’ names were missing in the original version of the authorship of the paper [1]. 

The two authors are Markus Kaller and Heiko Hermeking, who should be listed as the sixth and seventh authors. Their affiliations are (Markus Kaller ^5^, Heiko Hermeking ^5,6,7^):

^5^  Experimental and Molecular Pathology, Institute of Pathology, Ludwig-Maximilians-University Munich, 80337 Munich, Germany; markus.kaller@med.uni-muenchen.de (M.K.); heiko.hermeking@med.uni-muenchen.de (H.H.)
^6^  German Cancer Consortium (DKTK), Partner Site Munich, 80336 Munich, Germany
^7^  German Cancer Research Center (DKFZ), 69120 Heidelberg, Germany

The “Author Contributions” statement thus should be updated to the following version:

**Author Contributions:** Conceptualization, R.K.; Data curation, A.E.W. and T.S.; Formal analysis, A.E.W., T.S., B.H., and R.K.; Funding acquisition, R.K.; Investigation, A.E.W. and R.K.; Methodology, A.E.W., M.K., H.H., and R.K.; Project administration, R.K.; Resources, B.H., C.V., I.S., M.K., H.H., and D.v.S.; Software, T.S.; Supervision, R.K.; Validation, R.K.; Visualization, A.E.W.; Writing—original draft, A.E.W. and R.K.; Writing—review and editing, A.E.W., I.S., D.v.S., and R.K. All authors have read and agreed to the published version of the manuscript. 

We apologize for this error and state that the scientific conclusions are unaffected. The original article has been updated.
