# Supplementary material for: SP8 Promotes an Aggressive Phenotype in Hepatoblastoma via FGF8 Activation
Source: Cancers (Basel). 2020 Aug 15;12(8):2294. doi: 10.3390/cancers12082294 (PMC7465460; doi:10.3390/cancers12082294)
Supplement: Supplementary file 1 [file cancers-12-02294-s001.zip › cancers-859260 supplement figures and tables/cancers-859260 -Original Blots.pptx]

## Slide 1
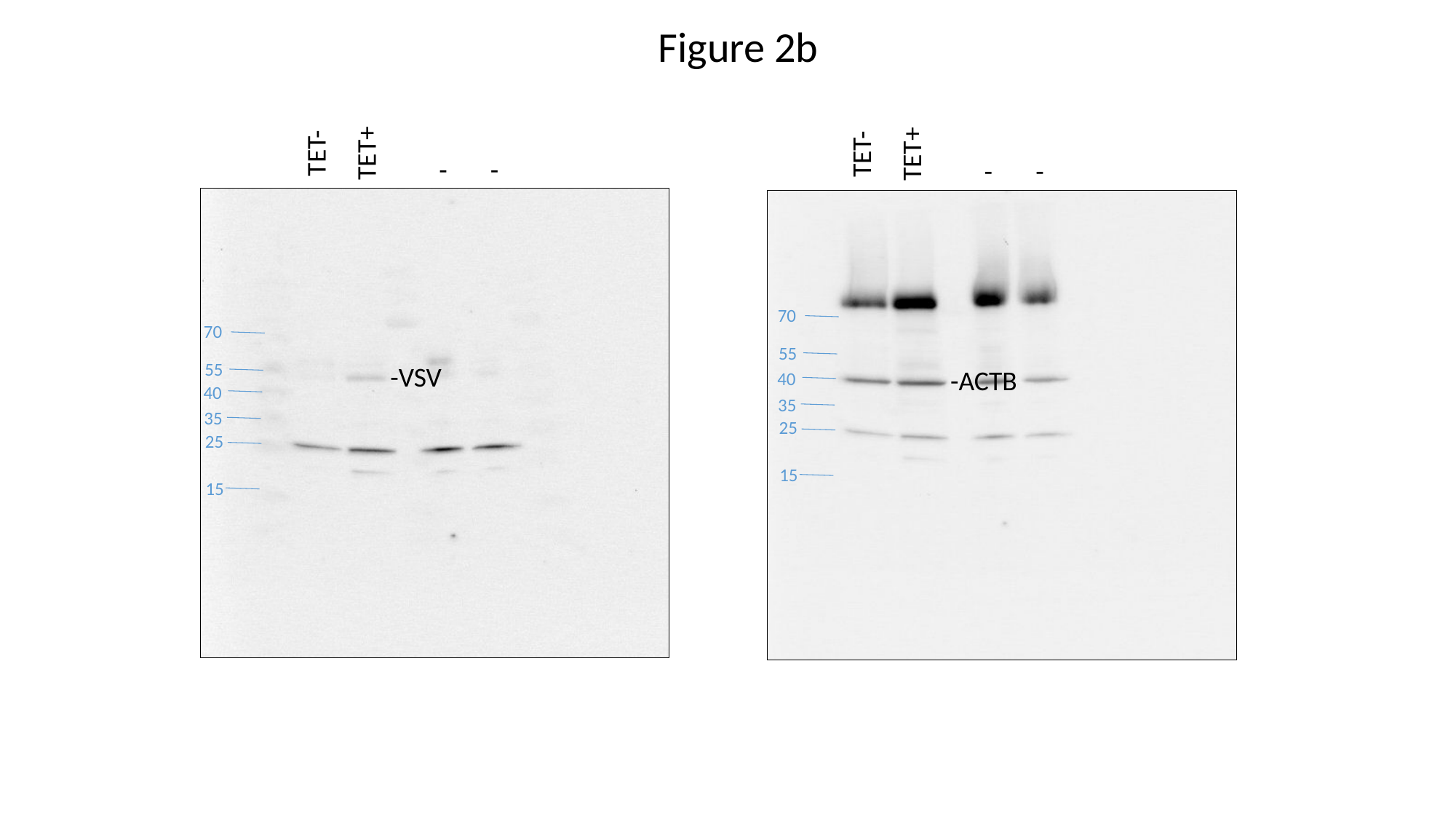

Figure 2b
TET-
TET+
TET-
TET+
- -
- -
70
70
55
55
-VSV
-ACTB
40
40
35
35
25
25
15
15

## Slide 2
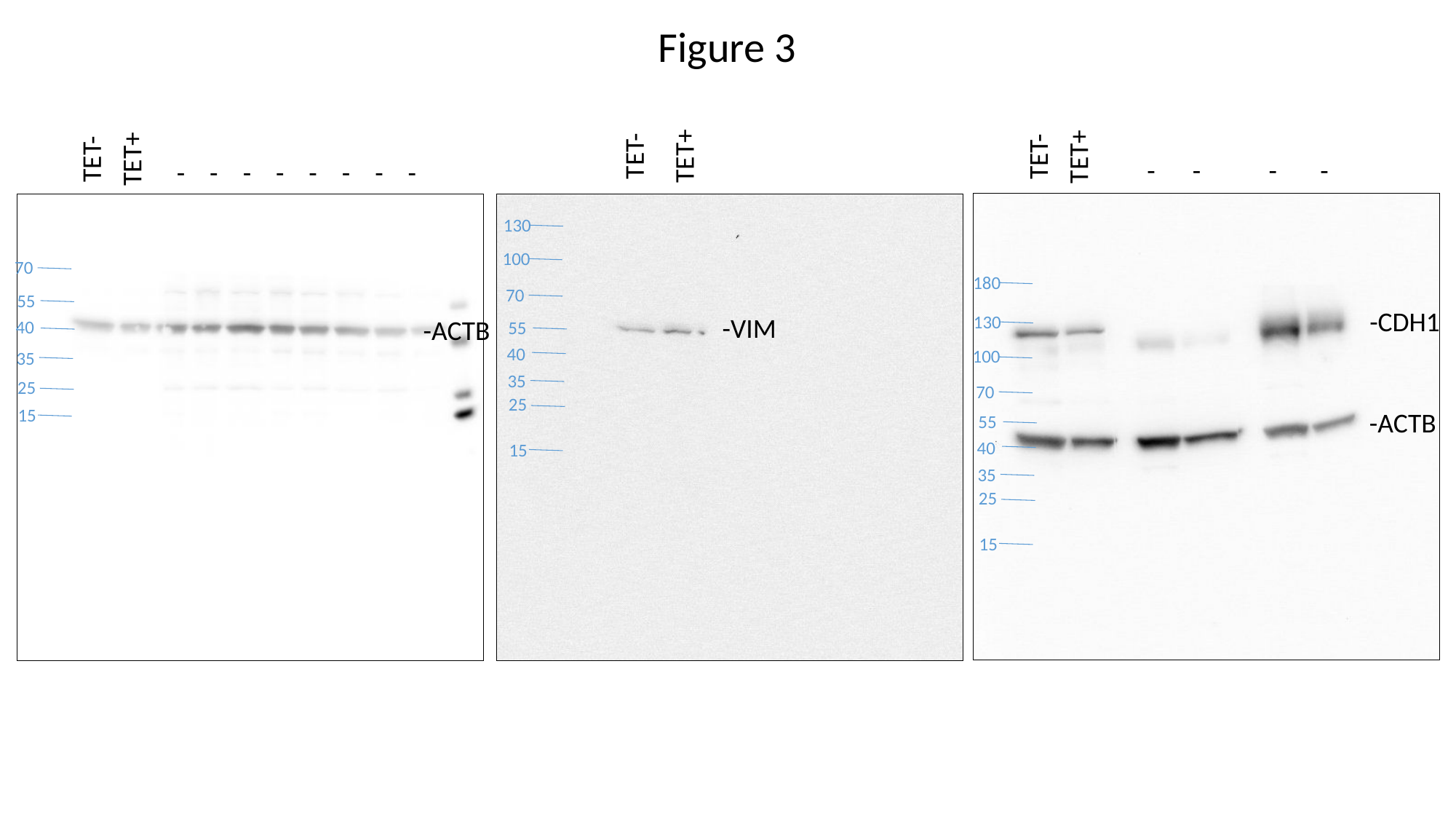

Figure 3
TET-
TET+
TET-
TET+
TET-
TET+
- - - -
- - - - - - - -
130
100
70
180
70
55
-CDH1
130
-VIM
-ACTB
40
55
40
100
35
35
25
70
25
15
-ACTB
55
40
15
35
25
15

## Slide 3
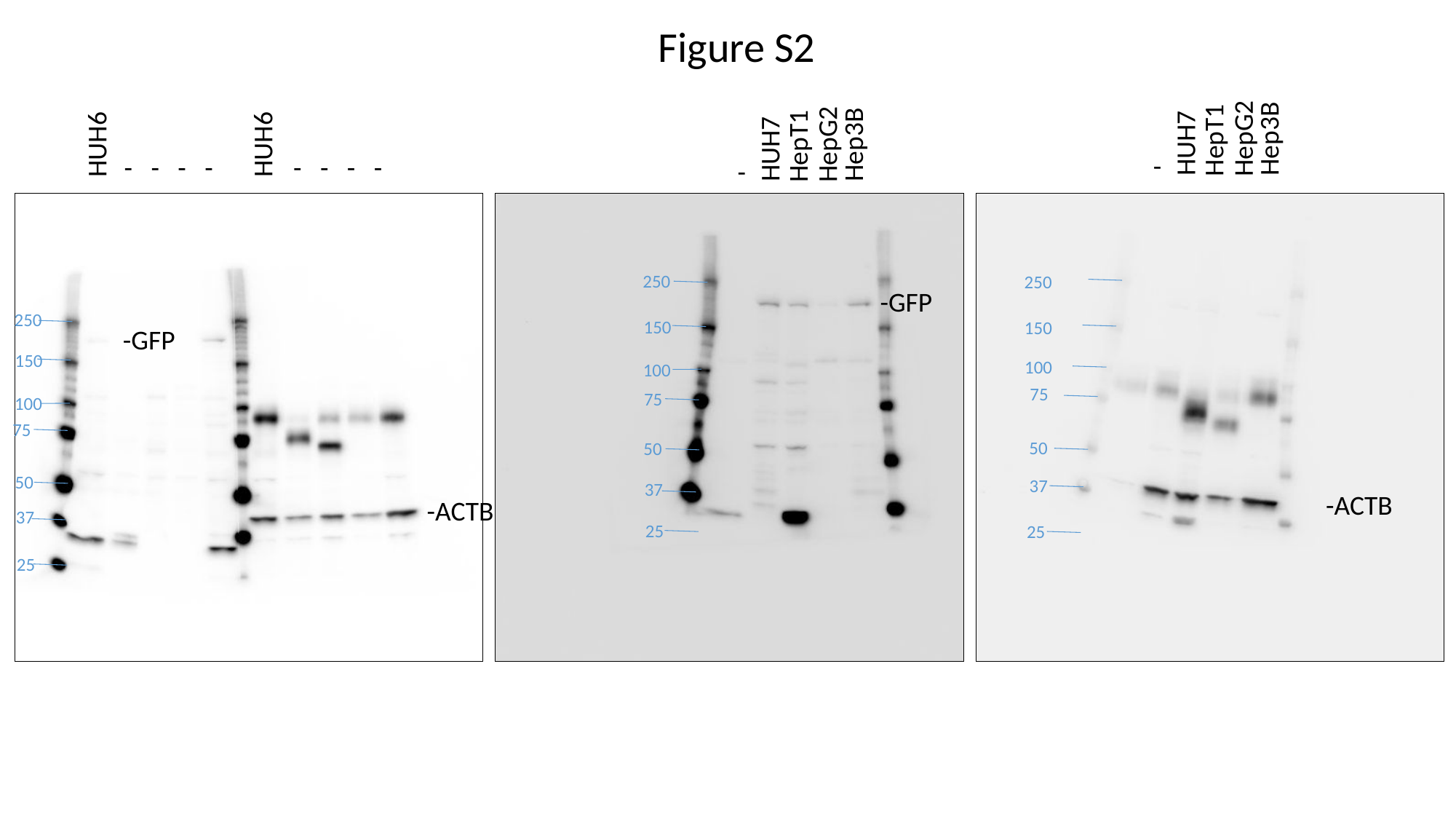

Figure S2
HepG2
Hep3B
HepT1
HUH7
HepG2
HUH6
HUH6
Hep3B
HepT1
HUH7
-
- - - -
- - - -
-
250
250
-GFP
250
150
150
-GFP
150
100
100
75
75
100
75
50
50
50
37
37
-ACTB
-ACTB
37
25
25
25
