# Supplementary material for: SP8 Promotes an Aggressive Phenotype in Hepatoblastoma via FGF8 Activation
Source: Cancers (Basel). 2020 Aug 15;12(8):2294. doi: 10.3390/cancers12082294 (PMC7465460; doi:10.3390/cancers12082294)
Supplement: Supplementary file 1 [file cancers-12-02294-s001.zip › cancers-859260 supplement figures and tables/cancers-859260 SM update.pdf]

*Supplementary Materials*

# SP8 Promotes an Aggressive Phenotype in Hepatoblastoma via FGF8 Activation

**Alexandra Elisabeth Wagner, Thomas Schwarzmayr, Beate Häberle, Christian Vokuhl, Irene Schmid, Dietrich von Schweinitz and Roland Kappler**

## Generation of Constructs

To generate **pcDNA3-SP8-VSV**, the SP8 cDNA (NM_182700) was PCR-amplified from pCMV/Tag2B/FLAG/SP8 (kindly provided by Raul Urrutia, Mayo Clinic, Rochester, NY, USA) by using two specific primers containing restriction sites for BamHI and EcoRI (Primer #1, Table S2). The EcoRI and BamHI-digested PCR fragment was then cloned into the pcDNA3-VSV vector (kindly provided by Heiko Hermeking, LMU Munich, Germany).

For constructing the episomal **pRTR-SP8-VSV**, the SP8-VSV ORF was released from pcDNA3-SP8-VSV via BamHI and NheI restriction sites, ligated into pUC19-Sfil shuttle vector (kindly provided by Heiko Hermeking, LMU Munich, Germany), isolated with Sfil, and finally ligated into pRTR (kindly provided by Heiko Hermeking, LMU Munich, Germany).

For cloning the all-in-one vector **pPlat-dCas9-GFP-KRAB**, pPlat-gRNA2-TET1 (from addgene, #82559) was used as a backbone and subjected to restriction enzyme digestion via BamHI to remove the sun tag. The KRAB domain was PCR-amplified from pLKO5d.SFFV.dCas9-KRAB.P2A.BSD (a kind gift from Jan-Henning Klusmann, University Hospital Halle, Germany) by using two specific primers containing restriction sites for NheI and NotI (Primer #2, Table S2) and subsequently cloned into pPlat-gRNA2-TET1 (without sun tag). GFP-KRAB was additionally PCR-amplified from dCas9-GFP-KRAB with two specific primers both containing the BamHI restriction site (Primer #3, Table S2) and cloned back into pPlat-dCas9-GFP-KRAB.

The empty all-in-one control vector **pPlat-dCas9** was obtained by BamHI digestion of pPlat-gRNA2-TET1, followed by religation.

For the CRISPR-Cas9 interference approach, guide RNAs (gRNA; Table S2) were inserted into pPlat-dCas9-GFP-KRAB or pPlat-dCas9 using the NEBuilder® HiFi DNA Assembly kit (New England Biolabs, Ipswich, MA, USA) according to manufacturer's instructions. In brief, 10 nmol gRNAs (Table S2) were first annealed by 4 cycles consisting of 5 sec denaturation at 98 °C, primer annealing for 15 sec at 64–72°C (temperature was individually determined using the $T_m$ Calculator Tool provided by Thermo Fisher), and extension for 15 sec at 72 °C. Annealed primers were then mixed with HiFi DNA assembly Master Mix and the AflI-linearized vectors at a molar ratio (vector:insert) of 6:1 to a total amount of fragments of 0.2 pmol and incubated at 50 °C for 1 h. The generated constructs are mentioned below in the CRISPR-Cas9 interference testing and optimization section.

All vectors were subsequently Sanger sequenced for verification using the following primers (Table S2): T7 and SP6 for pcDNA3-SP8-VSV, SP8 qPCR for pRTR-SP8-VSV, pPlat-KRAB_seq1 and pPlat-KRAB_seq2 for pPlat-dCas9-GFP-KRAB, and U6 for the gRNA constructs.

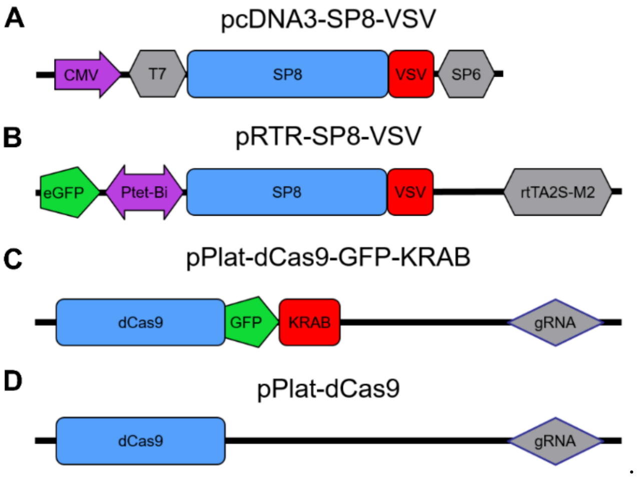


**Figure S1.** Schematic overview of the generated constructs. (**A**) pcDNA3-SP8-VSV, (**B**) pRTR-SP8-VSV, (**C**) pPlat-dCas9-GFP-KRAB and (**D**) pPlat-dCas9. CMV, cytomegalovirus promoter; T7, T7 promoter; SP8, SP8 cDNA; VSV, VSV-G-tag; SP6, SP6 promoter; eGFP, enhanced green fluorescent protein; Ptet-Bi, bidirectional tetracyclin inducible promoter; rtTA2S-M2, reverse tet transactivator 2S-M2; dCas9, deactivated CRISPR associated protein 9; KRAB, Krüppel-associated box repressor domain; gRNA, guide RNA.

## Transfection with Plasmid-DNA and SiRNA

For plasmid-DNA transfection, 1 µg plasmid in 50 µL water was mixed with 3.3 µL of FuGENE® HD transfection reagent and incubated for 10 min at room temperature. Then, the mixture was added to $1 \times 10^5$ cells/946.7 µL of cell suspension, shacked at 37 °C and 800 rpm for 7 min and transferred to a 6-well plate for overnight incubation at 37 °C.

SiRNA transfection was carried out using the EPI2500 device (Dr. L. Fischer, Laborgeräte Fischer, Heidelberg, Germany) for electroporation. To do so, 200 nM siRNA for SP8 or non-targeting siRNA (Dharmacon, Lafayette, CO, USA) were added to $2 \times 10^6$ cells/300 µL in a cuvette and pulsed at 350 V (HUH6, HUH7, Hep3B) or 250 V (HepT1, HepG2) for 10 msec.

## Validation of the pPlat-dCas9-GFP-KRAB Construct

To proof successful cloning, pPlat-dCas9-GFP-KRAB was transfected into the five hepatoma cell lines HUH6, HUH7, HepT1, HepG2 and Hep3B and the fusion protein dCas9-GFP-KRAB detected via Western blotting using an anti-GFP-antibody (Novus biologicals, Littleton, CO, USA), as shown in Figure S2.

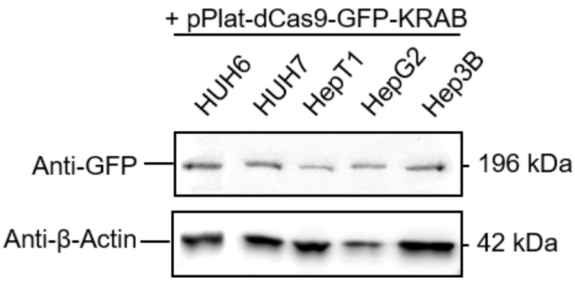


**Figure S2.** Detection of dCas9-GFP-KRAB fusion protein using anti-GFP-antibody in five hepatoma cell lines 48 h after transfection by Western blotting. Immunodetection of β-actin was used to standardize for equal protein loading.

## Expression Analysis of Liver Tumor Cell Lines

To determine appropriate cell lines for overexpression and knock-down experiments, the endogenous *SP8* and *FGF8* expression was determined by Q-PCR as indicated in Figure S3.

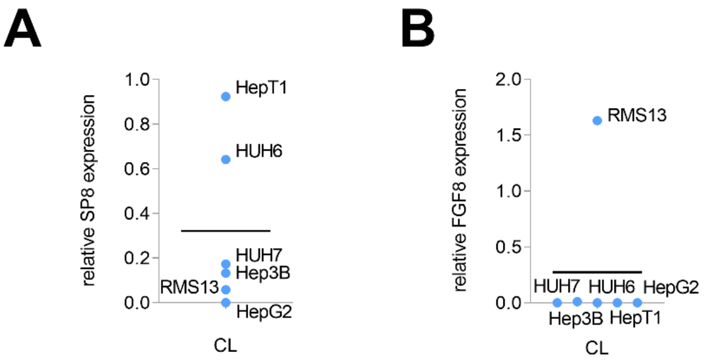


**Figure S3.** Endogenous SP8 (**A**) and FGF8 (**B**) expression levels in HepT1, HUH6, HUH7, Hep3B, HepG2 and RMS13 cells measured by Q-PCR and normalized to the house-keeping gene *TBP*.

To analyze successful SP8 modulation and subsequent expression changes of its target gene *FGF8*, *SP8* and *FGF8* expression levels were determined by Q-PCR as indicated in Figure S4.

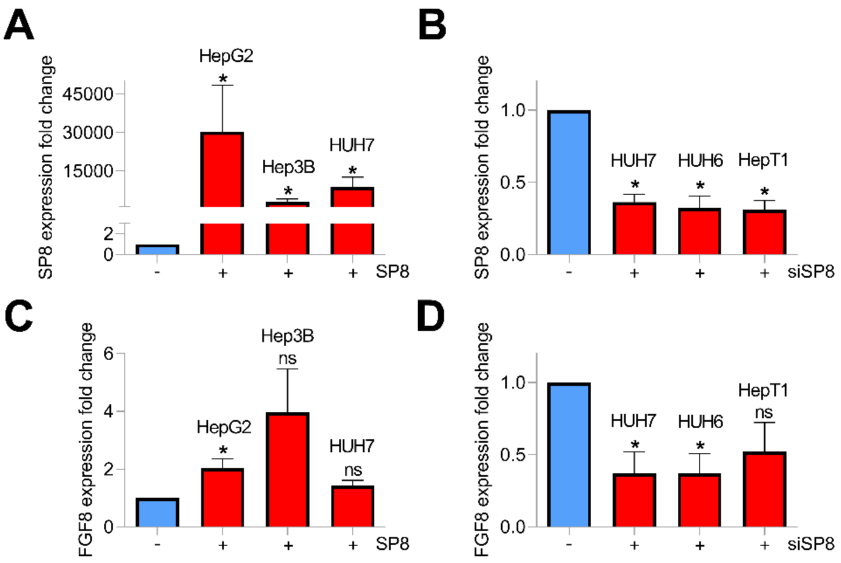


**Figure S4.** Gain- and loss-of-function expression studies in liver cancer cell lines after transfection with either (**A**, **C**) an empty control vector (−SP8) and a SP8 cDNA containing expression plasmid (+SP8), or (**B**, **D**) non-targeting siRNAs (siNTC) and a siRNA directed against *SP8* (siSP8), respectively. (**A**, **B**) *SP8* expression was measured 24 h after transfection by Q-PCR and normalized to the house-keeping gene *TBP*. Means ± SEM were calculated from four independent experiments. (**C**, **D**) *FGF8* expression was measured 48 h after transfection by Q-PCR and normalized to the house-keeping gene *TBP*. Means ± SEM were calculated from three independent experiments. (*$p < 0.05$)

To monitor the effects of the inhibitor Mithramycin A on *SP8* and *FGF8* expression levels, their expression level changes were determined by Q-PCR as indicated in Figure S5.

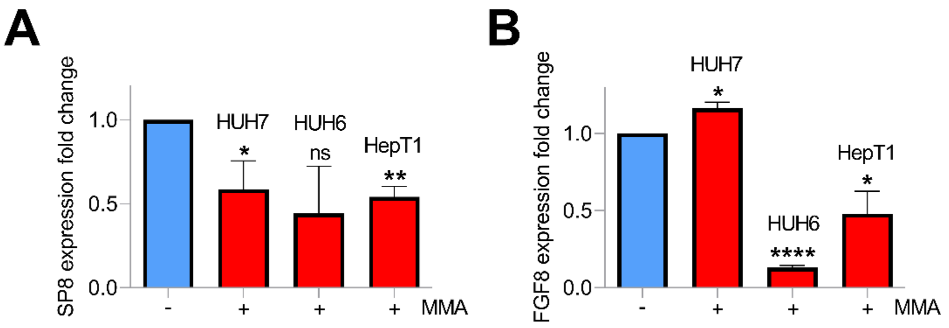


**Figure S5.** RNA expression of (**A**) *SP8* and (**B**) *FGF8* was determined 48 h after MMA treatment using Q-PCR and normalized to the house-keeping gene *TBP*. Means ± SEM were calculated from two independent experiments. (*p < 0.05, ** p < 0.01, *** p < 0.001, ****p < 0.0001)

## CRISPR-Cas9 Interference Testing and Optimization

To validate the performance of the pPlat-dCas9-GFP-KRAB vector in the CRISPR-Cas9 interference approach, a gRNA (gRNA-CMV; Table S2) directed against the transcription start site of the CMV promoter was designed and cloned into the vector system as described above.

After transient transfection of five hepatoma cell lines with 4 µg pPlat-dCas9-GFP-KRAB-gRNA-CMV (control cells with pPlat-dCas9-gRNA-CMV), 4 µg of the reporter plasmid pRL-CMV (Promega, Madison, WI, USA), and 0.8 µg of the normalization plasmid pGli-TK for 72 h, luciferase activity was measured using the Dual Luciferase Assay System kit according to the manufacturer's protocol (Promega). Relative luciferase activity (in arbitrary units) was calculated as fold induction after normalization for transfection efficiency. This experiment clearly indicated that the pPlat-dCas9-GFP-KRAB-gRNA-CMV construct was able to significantly suppress the CMV-driven expression of the luciferase reporter (Figure. S4A).

To establish a robust FGF8 knock-down, RMS13 cells were taken, as they showed the highest FGF8 expression (Figure S3B). We tested a total of five different gRNAs targeting FGF8 around its transcription start sites. With gRNA-1 (Table S2) we were able to reach a knock-down of 78% in RMS13 cells (Figure S4B), which was chosen for further experiments in Hep3B pool cells.

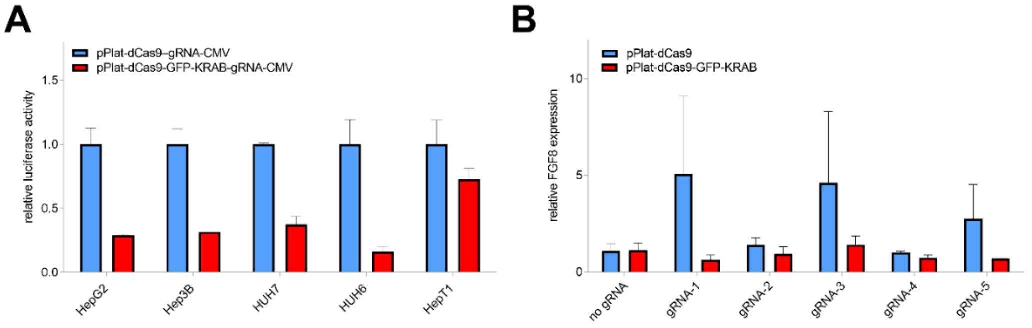


**Figure S6.** (**A**) Bar graph showing results of the luciferase assay with an inhibition of luciferase activity in HepG2, Hep3B, HUH7, HUH6 and HepT1 cells using pPlat-dCas9-GFP-KRAB-gRNA-CMV compared to pPlat-dCas9–gRNA-CMV. (**B**) Bar graphs indicating FGF8 knock-down in RMS13 cells with five different gRNA directed against FGF8 and an empty vector control.

© 2020 by the authors. Licensee MDPI, Basel, Switzerland. This article is an open access article distributed under the terms and conditions of the Creative Commons Attribution (CC BY) license (http://creativecommons.org/licenses/by/4.0/).
